# Spatial and Temporal Distribution of Multiple Cropping Indices in the North China Plain Using a Long Remote Sensing Data Time Series

**DOI:** 10.3390/s16040557

**Published:** 2016-04-19

**Authors:** Yan Zhao, Linyan Bai, Jianzhong Feng, Xiaosong Lin, Li Wang, Lijun Xu, Qiyun Ran, Kui Wang

**Affiliations:** 1 Institute of Remote Sensing and Digital Earth, Chinese Academy of Sciences, Beijing 100094, China; zhaoyanjun_1230@sina.com (Y.Z.); wl_6887@163.com (L.W.); qiyunran123@sina.com (Q.R.); wkrealmadrid@hotmail.com (K.W.); 2 Key Lab of Agri-information Service Technology, Ministry of Agriculture of China, Agricultural Information Institute, Chinese Academy of Agricultural Sciences, Beijing 100081, China; 3 College of architecture and urban planning, Chongqing Jiaotong University, Chongqing 400074, China; lxsgis@163.com; 4 Institute of Agricultural Resources and Regional Planning, Chinese Academy of Agricultural Sciences, Beijing 100081, China; xulijun@caas.cn

**Keywords:** GLASS LAI, NCP, multiple cropping index, spatial and temporal changes, remote sensing

## Abstract

Multiple cropping provides China with a very important system of intensive cultivation, and can effectively enhance the efficiency of farmland use while improving regional food production and security. A multiple cropping index (MCI), which represents the intensity of multiple cropping and reflects the effects of climate change on agricultural production and cropping systems, often serves as a useful parameter. Therefore, monitoring the dynamic changes in the MCI of farmland over a large area using remote sensing data is essential. For this purpose, nearly 30 years of MCIs related to dry land in the North China Plain (NCP) were efficiently extracted from remotely sensed leaf area index (LAI) data from the Global LAnd Surface Satellite (GLASS). Next, the characteristics of the spatial-temporal change in MCI were analyzed. First, 2162 typical arable sample sites were selected based on a gridded spatial sampling strategy, and then the LAI information was extracted from the samples. Second, the Savizky-Golay filter was used to smooth the LAI time-series data of the samples, and then the MCIs of the samples were obtained using a second-order difference algorithm. Finally, the geo-statistical Kriging method was employed to map the spatial distribution of the MCIs and to obtain a time-series dataset of the MCIs of dry land over the NCP. The results showed that all of the MCIs in the NCP showed an increasing trend over the entire study period and increased most rapidly from 1982 to 2002. Spatially, MCIs decreased from south to north; also, high MCIs were mainly concentrated in the relatively flat areas. In addition, the partial spatial changes of MCIs had clear geographical characteristics, with the largest change in Henan Province.

## 1. Introduction

**Figure 1:**
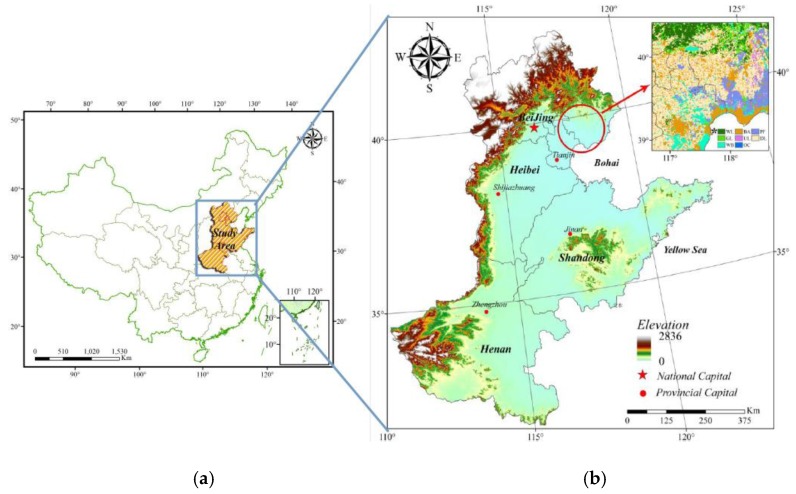
Location and scope of the study area. (**a**) Location of the study area within China; (**b**) map of the study area, with a regional inset map showing the study area within eastern China. (*BA*, Build-up Areas; *DL*, Dry Land; *GL*, Grassland; *OC*, Ocean; *PF*, Paddy Field; *WB*, Water Body; *WL*, Woodland; *UL*, Unused Land).

Agricultural areas are very sensitive to climate change and variability, which can lead to profound changes in regional agricultural resources and production [1,2]. The North China Plain (NCP; Figure 1), located in the mid-latitudes, serves as a major grain-cotton production region in China; more than one fifth of China’s food crops acreage and yield comes from here [3,4]. Therefore, the spatial and temporal variations of the cropping system, the characteristics of grain production structures, and the distribution of agricultural production of this region should be analyzed as should laws governing land management [5,6].

Humans need farmland to survive [7,8] and it plays a major role in food production and food security. Multi-cropping is an efficient and commonly used cropping system that relieves the pressure created by the limited availability of farmland resources while increasing crop output and farm income [9,10]. Cropping intensity is often measured using a multiple cropping index (MCI); an MCI measures the planting frequency of crop(s) in the same farmland in one year [11]. An MCI can reflect the ratio of use of water, soil, light energy, and other natural resources [12,13]. Therefore, MCI monitoring is used during the analysis and evaluation of the rational use of land (especially farmland) resources; this provides information related to food production, food security assessment, and scientific planning of agricultural development [14,15].

Currently, using long time serial satellite remote sensing data, the measurement of MCI and an analysis of its characteristics provide a relatively accurate and efficient analysis method. This method can be used to probe the effects of climate change on the dynamic spatial and temporal changes in agricultural production, especially when compared to traditional statistical methods [16,17,18]. Periodic growth curves of crops can be fitted, and MCIs can be easily extracted by building the serial vegetation indices covering a long period of time. These types of indices include the normalized difference vegetation index (NDVI), enhanced vegetation index (EVI) and leaf area index (LAI) [19,20]. These indices can be used with various denoising methods, e.g., curve fitting [21], harmonic analysis of time series (HANTS) filtering [22], Savitzky-Golay filtering [23,24] and Whittaker smoothing [25]. Many studies have been conducted with MCI monitoring using NDVI and EVI time series data derived from remote sensing data with moderate and low spatial resolution [26]. For example, Canisius *et al.* studied the cropping systems in Asia by employing a Fourier Transform (FT) and Decision Tree (DT) method based on NOAA’s Advanced Very High Resolution Radiometer (NOAA/AVHRR) NDVI data [27]. In addition, Galford *et al.* and Sakamoto *et al.* used a Smooth Wavelet Smoothing algorithm to denoise and reconstruct the moderate-resolution imaging spectroradiometer (MODIS) EVI dataset, and then monitored multi-cropping information in Brazil and in the Vietnam Mekong region, respectively [28,29]. Also, Li *et al.* and Yang *et al.* extracted the MCIs of two areas of Shanxi Province, China, from 2000 to 2007 and the Bohai Rim in China for 2000, 2004, and 2008 using the SPOT/VEGETATION (VGT) NDVI time serial data, respectively [30,31]. The MODIS NDVI and EVI time series were used to monitor the MCIs of farmland at different regional scales [32,33,34,35].

The use of remote sensing data to map cropping systems often focuses on directly mapping the distribution of cropping patterns based on multi-spectral data [36]. For instance, Martínez-Casasnovas *et al.* used a Geographic Information System (GIS) with overlay analysis to map the main multi-year cropping patterns in the Flumen Irrigation District. This analysis was based on a 7-year time-series of cropping images that had been derived from the supervised classification of Landsat-5 Thematic Mapper and Landsat-7 and Enhanced Thematic Mapper+ imagery [37]. Toshihiro *et al.* analyzed the phenological characteristics of rice growing areas of the Mekong Delta, Vietnam, using MODIS EVI time series products, and documented the five main rice planting patterns [28]. Yang *et al.* used NOAA-AVHRR-NDVI (of 1986 and 1996) and SPOT-VEGETATION-NDVI (of 2000) data to map the main cropping systems of China [33]. However, they used very dense samples and spatial interpolation methods (e.g., Kriging and Inverse Distance Weighting approaches) to map the spatio-temporal distribution of cropping systems (or multiple crop indices). This technique can reduce mapping errors resulting from the effects of many mixed pixels that are often found in remote sensing images, especially on a very segmented area [38,39]. In this paper, we employed a Kriging spatial interpolation method in terms of the variation law of the parameter (MCI) to map the spatio-temporal distribution information of MCIs of dry land in the NCP from 1982 to 2012 and to analyze their characteristics. In some subareas this landscape exhibits very high levels of fragmentation.

Meanwhile, few research studies have reported on the extraction of MCIs related to farmland based on LAI time series derived from remote sensing data. This parameter, LAI, provides a very useful structural vegetation index that is defined as the single-sided leaf area in per unit ground area. When compared with the other vegetation indices such as NDVI and EVI, LAI can well represent the characteristics of the vegetation canopy as well as accurately and quantitatively reflect crop growth information [20]. Hence, in this paper, the MCIs of dry land in the NCP were extracted from the Global LAnd Surface Satellite (GLASS) LAI time series products collected during 1982–2012, and subsequently, the spatial-temporal characteristics of the MCIs were analyzed.

## 2. Material and Methods

### 2.1. Study Region

The NCP of Northern China forms one of the largest plains in eastern Asia. Most of the NCP is less than 50 meters above sea level, and serves as one of the major food-producing regions in China. It extends over the municipalities of Beijing and Tianjin, and includes much of Henan, Hebei, and Shandong provinces, as well as parts of northern Jiangsu and Anhui provinces; these last two provinces are not included in this study. The studied ranged across 31.6°N–42.5°N and 110.4°E–122.6°E (Figure 1). The flat, open typical fluvial NCP has many rivers and lakes, and is composed of very deep alluvial deposits, principally re-deposited loess resulting in fertile soil. Most of its area experiences a warm temperate continental monsoon climate with four distinct seasons, and features relatively abundant light and heat resources. The annual average temperature is 12–15 °C), with an annual accumulated temperature (≥0 °C) of 4100–5400 °C, and an annual frost-free period of 190–220 days. This climate can meet the needs of a double cropping system within one year and/or triple cropping system over two years. The average annual precipitation of 500–1000 mm is unevenly distributed over time, and is largely concentrated from July to September. Most of the dryland farmland of the NCP supports a typical cropping practice of mainly rotationally cultivated winter wheat and summer corn. However, farmers may grow other crops such as corn, sorghum, peanuts, vegetables, and cotton and the region exhibits a high degree of regional agricultural development [40].

### 2.2. Data

The GLASS LAI product, a global LAI product with long time series, has a temporal resolution of eight days and is available from 1982 to 2012. This product is archived in a hierarchical data format for NASA’s Earth Observing System. The Center for Global Change Data Processing and Analysis at Beijing Normal University in China generated and released this data. The LAI product (1982–1999) with a spatial resolution of 0.05° × 0.05° was generated from AVHRR reflectance, and the MODIS data (2000–2012), with the spatial resolution of 0.05° × 0.05° and 1 × 1 km, and is derived from MODIS land surface reflectance (MOD09A1) [41,42]. This study used the GLASS 0.05° resolution data. The product has very good accuracy in space and time because it was produced under a strict quality control system. The product has remarkable advantages related to the integrity of spatial scope and continuity of the time series. In addition, any pixels with cloud and snow have been removed from the images. Missing values were extrapolated, and the images were processed using optimized filters, which can reduce the errors of the LAI product to meet the needs of highly efficient applications [43,44]. The LAI digital number (DN) values range from 0–255 in the image of the product, setting a valid value range of 0–100. Meanwhile, the other values are set to 255 and are deemed to be water-filled areas; a DN value of an image is converted into a LAI value by the formula: LAI = DN × scale factor + add offset with scale factor = 0.1 and add offset = 0.

The land use dataset was derived from China’s land use remote sensing monitoring database in 2010 at a 1:100,000 scale, provided by the Science Data Center of Resources and Environment, Chinese Academy of Sciences. The data were produced through artificial visual interpretation based on Landsat Thematic Mapper and Enhanced Thematic Mapper satellite images, which include 25 secondary types with six primary types: farmland, woodland, grassland, water body, build-up areas, and unused land [45]. We used the land use data to extract the location and area information related to dry land, *i.e.*, arable land with a secondary type, over the NCP as defined above. Then, based on the obtained dry land information, we monitored the MCIs of the NCP and fulfilled the extension of spatial scale for the MCIs (mapping MCIs).

Additionally, this study used farming season and agricultural statistical data for dry land of the NCP. These data were provided by the Department of Planting Industry Management, Ministry of Agriculture of the P. R. of China. The study also employed agricultural statistical data, including the dry land area and total sown area of crops for the study region. These data were obtained from the *China Statistical Yearbooks* for 1982, 1987, 1992, 1997, 2002, 2007, and 2012 [46]. These data were used to calculate the statistical multi-cropping indices of each subarea and the total study area, and to comparatively analyze and validate the MCI monitoring results using the previously discussed remote sensing methods [10,12,47].

### 2.3. Methods

**Figure 2:**
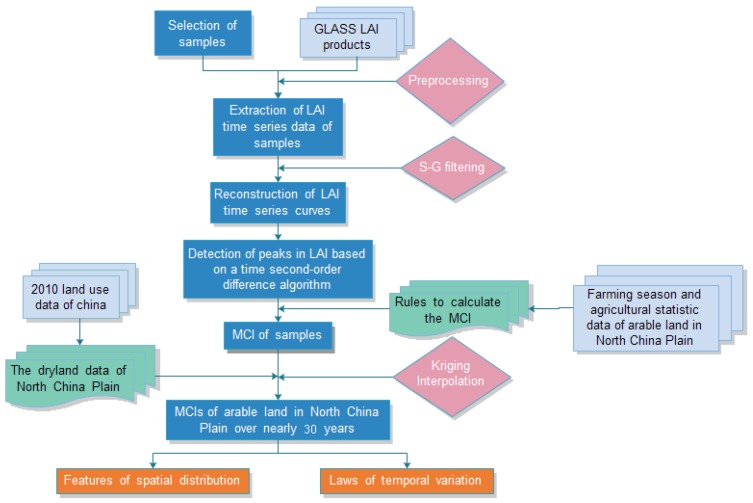
Schematic diagram of the extraction processes of multiple cropping indices (MCIs) of dry land. Note: LAI, leaf area index; GLASS, Global LAnd Surface Satellite.

Based on the reflectance spectra of green vegetation in remote sensing imagery, large-area LAIs can be retrieved and easily reveal the growth status and vegetation in an objective area. LAI time-series dataset can represent the intra-year or inter-annual growth characteristics of cultivated crops [48] and through using them, phenological features can be detected. During growth, a crop generally moves through seeding, emergence, jointing, heading, to harvest stages. Therefore, a crop’s LAI values will change from increasing to peaking (perhaps with multiple peaks) to declining. Obviously, a marked peak, two peaks, and three peaks exists in single-cropping, double-cropping and triple-cropping systems, demonstrating the associated dynamic changes of crop growth in each type of cropping system. This allows crop LAI time series curves to be constructed in areas with dry land farming, so that the number of peak(s) can be employed to extract the MCI information [49]. Based on the GLASS LAI long time series products, a scheme has been developed for extracting and analyzing the MCIs of dry land over the NCP in this study (Figure 2).

#### 2.3.1. Spatial Sampling and Extracting LAI Time Series Information

**Figure 3:**
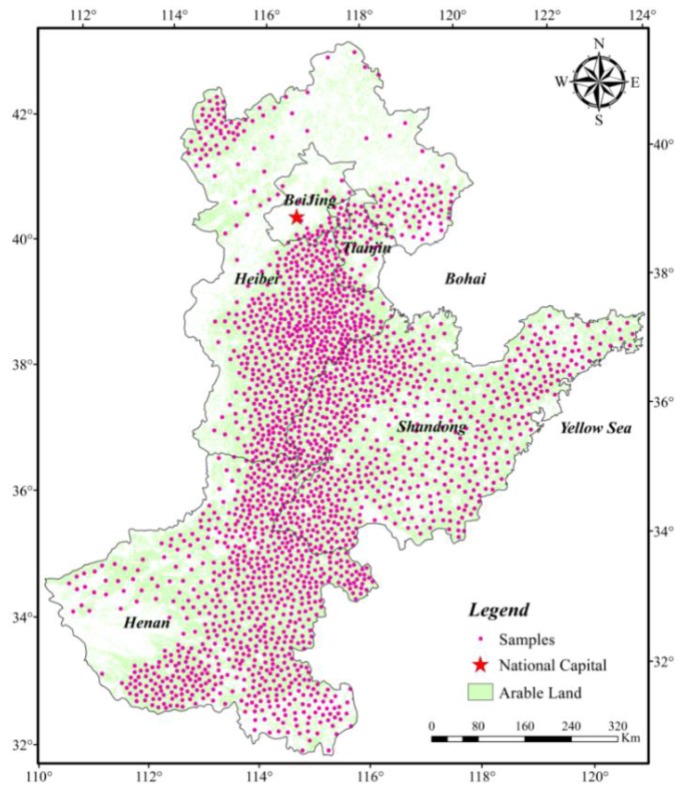
Distribution of dry land sample sites in the North China Plain.

The 2162 typical samples (Figure 3) were selected from high-definition, high-resolution imagery (e.g., Google Earth imagery with Version 6.1.0.5001, 2012) based on a gridded spatial sampling strategy where there were pure or nearly pure pixels of images within the study area. Then, we extracted the LAI information in each sample in 1982, 1987, 1992, 1997, 2002, 2007, and 2012 using the GLASS LAI products.

#### 2.3.2. Filtering and Reconstructing LAI Curves

**Figure 4:**
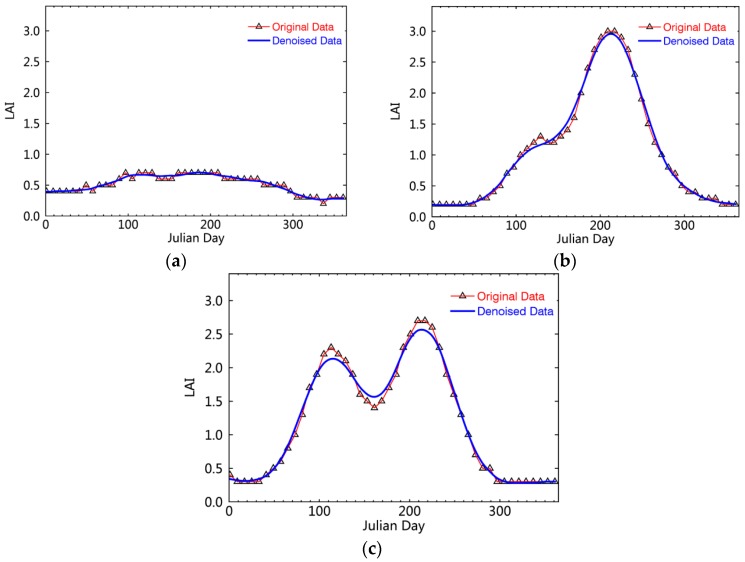
Smoothness of crop Leaf Area Index (LAI) time series curves in the North China Plain. (**a**) No-peak curve; (**b**) Unimodal curve; (**c**) Bimodal curve.

Using the GLASS LAI data directly, some “small” serrated peaks were observed in the obtained LAI time series curves of each sample. To avoid “pseudo” and “false” peak phenomena, the LAI change trendlines should be processed further with a denoising method to improve the usability of the data. Here, we used the Savizky-Golay (S-G) filtering method to remove the noise components of the LAI long time series of samples and reconstruct the LAI curves of crop growth [50]. The reconstructed LAI curves of crops have apparent peaks, which can more effectively and accurately present the multiple cropping situations (Figure 4). For example, Figure 4a shows the low LAI values of the curve for bare or vacant land with no obvious peaks (or troughs), while the single and double peaks in Figure 4b,c can be linked with dry land in a single- and double-cropping system, respectively.

#### 2.3.3. Extracting MCIs

Given that the LAI value on a reconstructed LAI time series curve in each sampling pixel location is a discrete function of the time phases of the GLASS LAI images, we employed a second-order difference algorithm to detect the number of the peaks for the reconstructed LAI curves, and further extract the MCIs of dry land from the NCP for 1982 to 2012 [51]:

(1) $$s_{i}^{\left(1\right)}=N_{LAI_{i}}-N_{LAI_{i-1}}$$

(2) $$s^{\left(2\right)}=\{\begin{array}{cc} 1 & s_{i}^{\left(1\right)}\geq 0\\ -1 & s_{i}^{\left(1\right)}<0 \end{array}$$

(3) $$s_{i}^{\left(3\right)}=s_{i}^{\left(2\right)}-s_{i-1}^{\left(2\right)}$$

where $N_{LAI_{i}}$ is the LAI value of a sampling pixel with *i* being the time series phase of GLASS LAI imagery and $s^{\left(1\right)}$, $s^{\left(2\right)}$ and $s^{\left(3\right)}$ are the correspondingly detecting parameters in Equations (1)–(3), respectively. Initially, the sequence $s^{\left(1\right)}$ was calculated by Equation (1), *i.e.*, using the differences between the LAIs of two adjacent time phases in each sampling pixel location. After the sequence $s^{\left(2\right)}$ was obtained by reassignment of element of $s_{i}^{\left(1\right)}$, a second difference was carried out by $s^{\left(2\right)}$, and then the sequence $s^{\left(3\right)}$ was obtained, which was composed of several numerical values (−2, 0 and 2). Hence, a value of −2 in $s^{\left(3\right)}$ could be regarded as linked to a wave peak on the crop growth curve of a sample where it is located in a position of the curve in accordance to the two adjacent values of 0 before and after it. In the same way, this was used to decide the corresponding positions of more than one peak on the crop growth curve of a sample [12,52].

Meanwhile, owing to the influence of image quality, mixed pixels and so on, the change curves of the LAI time series for partial samples would show up as noise peaks resulting from the occurrence of abnormal fluctuations. To remove the interference of pseudo peaks, the farming season and agricultural statistical data of dry land of the NCP were thus used to limit the minimum value and the time positions of peaks where they might appear in the LAI curves, and then the correct number of peaks that was validly extracted. Qualifications included:

(A) The time positions of peaks in the LAI curves of dry land appeared from 120 to 300 day of a year;
(B) The LAI value in the peaks could not be less than two under the single-peaked curve;
(C) The time differences between peaks could not be less than 40 days when multimodal peaks appeared in a LAI time series curve; in addition, the LAI value of the smaller peak could not be less than 40% of the maximum peak value.

Consequently, based on the relationship between the LAI time series curves of dry land and the MCIs, the calculation of the MCIs can easily be converted to the statistic parameter of number of peaks for the LAI curves as follows:

(4) $$MCI=F_{i}=\frac{\sum s_{j}(i)}{\sum p_{j}(i)}\times 100\%$$

where *MCI* represents the multiple cropping index; the $F_{i}$ represents the frequencies of wave peaks with the *i-th* year; $\sum s_{j}(i)$ is the amount of peaks for the curves of the LAIs of all samples during a year; $\sum p_{j}(i)$ is the total number of samples; and *j* (equal to 1, 2, 3,…) is the sample number.

#### 2.3.4. Mapping MCIs

Based on spatial characteristics of the MCI data of the dry land samples, different semivariogram models (*i.e.*, Gaussian, spherical, exponential, and linear models) were compared to select the most optimal Kriging model for spatial interpolation; then mapping of the MCIs of dry land in the NCP over nearly 30 years was conducted.

In the above steps, Interactive Data Language was applied to make it easier to interactively and visually preprocess the long time series remote sensing data and extract MCIs. Then, using ArcGIS software, the map sets of spatial distribution and time series of MCIs of dry land of the NCP were effectively produced.

#### 2.3.5. Validation Analysis

Using the two statistics of the dry land area ($T_{i}^{\left(j\right)}(real)$) and total sown area ($T_{i}^{\left(j\right)}(sown)$) of crops over the study administrative regions, derived from the China Statistical Yearbooks in 1982, 1987, 1992, 1997, 2002, 2007 and 2012, MCIs were calculated by formula (5) below:

(5) $$MCI_{i}(statistic)=\frac{T_{i}^{\left(j\right)}(sown)}{T_{i}^{\left(j\right)}(real)}\times 100\%$$

where $MCI_{i}(statistic)$ is the multiple cropping index in the *i-th* year with the number *j* (equal to 0, 1, 2, 3, 4, and 5) of the total area and six administrative regions mentioned above, respectively. Then, the results of the MCIs obtained by the previous remote sensing approach were comparatively analyzed and validated with the results from the statistical data [53].

## 3. Results

### 3.1. Temporal and Spatial Changes of MCI in the North China Plain

**Figure 5:**
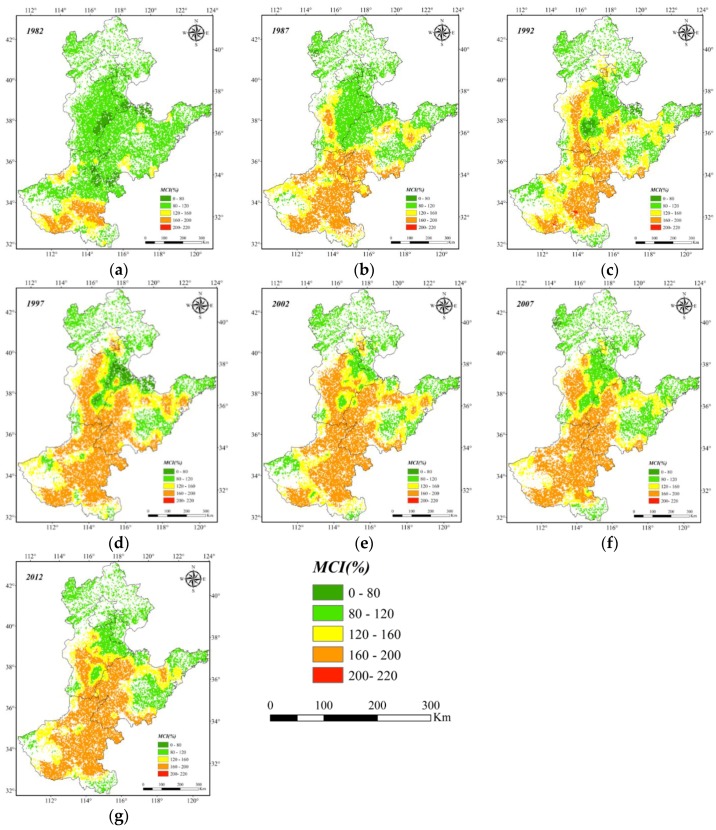
Distribution of Multiple Cropping Indices in the North China Plain from 1982 to 2012. (**a**) 1982; (**b**) 1987; (**c**) 1992; (**d**) 1997; (**e**) 2002; (**f**) 2007; (**g**) 2012.

The dry land MCIs of the NCP over the past 30 years were successfully extracted using Interactive Data Language codes that were combined with ArcGIS tools; then, the temporal-spatial pattern characteristics of the MCIs were obtained. The temporal and spatial changes of the MCIs in the NCP over the past 30 years exhibit a south to north decreasing spatial pattern (Figure 5). The dry land MCIs of the southern subregions with better light-heat and precipitation conditions (such as Henan) significantly exceed those of the northern parts (such as northwestern Hebei) that have relatively smaller values for those factors.

**Figure 6:**
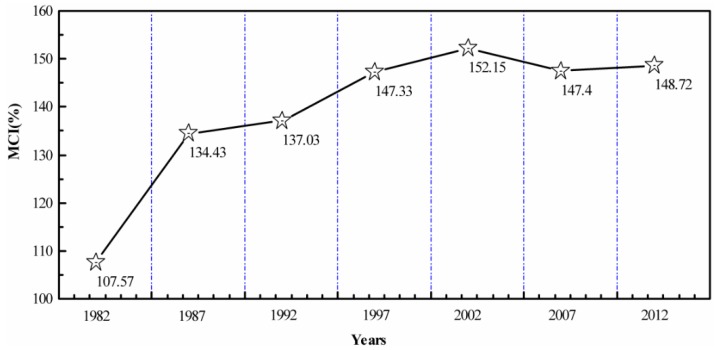
Variation tendency of the Multiple Cropping Indices in the North China Plain from 1982 to 2012.

The subregions with MCIs no less than 160% are mainly distributed in northern Henan, western Shandong and southern Hebei which are traditional and important national agriculture development regions [54]. The subregions with higher MCIs are mainly located in relatively flat areas such as the plains in Henan and Shandong provinces. However, the subregions with lower MCIs are mainly distributed in the densely populated and hilly areas with an arid climate [55], including Beijing, Tianjin and mountainous areas in Hebei Province. The variation tendency chart on the temporal scale of MCI in the NCP (Figure 6) was derived from the average MCIs in 1982, 1987, 1992, 1997, 2002, 2007, and 2012. The results show that the MCIs in the NCP had generally continued to increase during the past 30 years. The lowest value of the MCI, 107.57%, appeared in 1982, but the highest value of 152.15% was in 2002. The MCIs significantly increased during the period from 1982 to 2002 (with the largest increase from 1982 to 1987), followed by a slight decline from 2002–2012.

**Figure 7:**
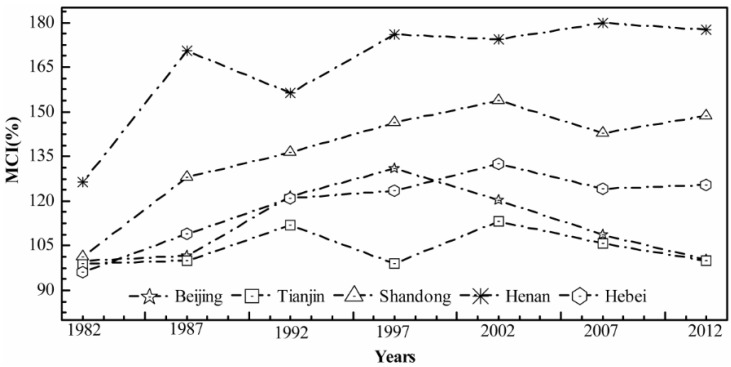
Multiple Cropping Indices in five subareas of the North China Plain from 1982 to 2012.

In contrast with the district level (Table 1 and Figure 7), the MCIs of dry land for each province also represented the same growth trend. For example, in Henan Province the MCI varied from 177.69% in 2012 to 126.44% in 1982, and had peaked at 179.95% in 2007. However, the MCIs of Beijing and Tianjin remained at a low level with minor variation.

**Figure 8:**
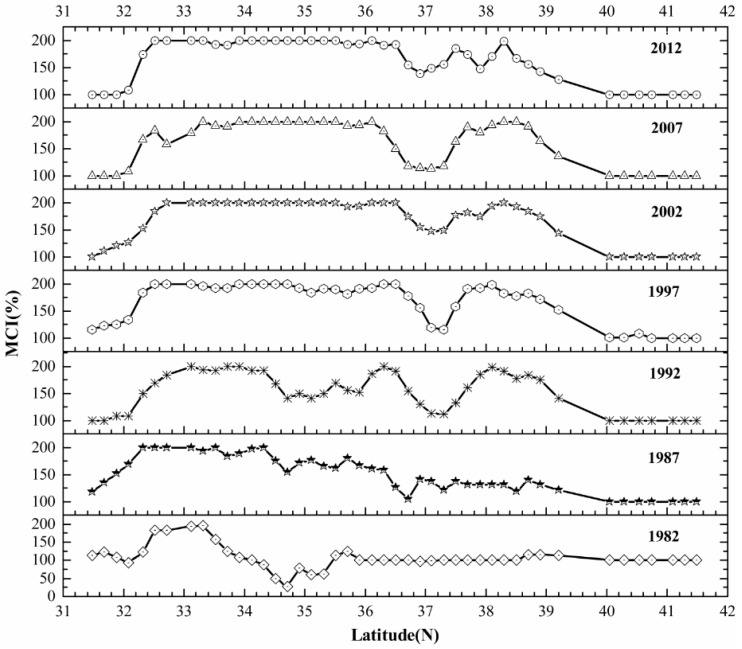
Multiple Cropping Indices of the North China Plain in the longitude of 115°E from 1982 to 2012.

The dry land MCIs were extracted longitudinally along the 115°E Meridian in the NCP over the past 30 years. The MCIs approximately dip from lower latitudes to higher latitudes (Figure 8). The MCIs in the zones from 31.4°N to 32°N, 36.7°N to 37.3°N and 40°N to 41.6°N are relatively lower because of the large area of mountains and hills in these regions (Figure 1). Conditions here led to poorer agricultural conditions and thus multi-cropping has failed to improve crop production over time. In contrast, the MCIs in flat areas from 32.2°N to 36.2°N and 37.6°N to 38.6°N tend to be relatively higher, especially after 1997, and tend to remain relatively stable, including a maximum MCI above 160% in some subregions.

**Figure 9:**
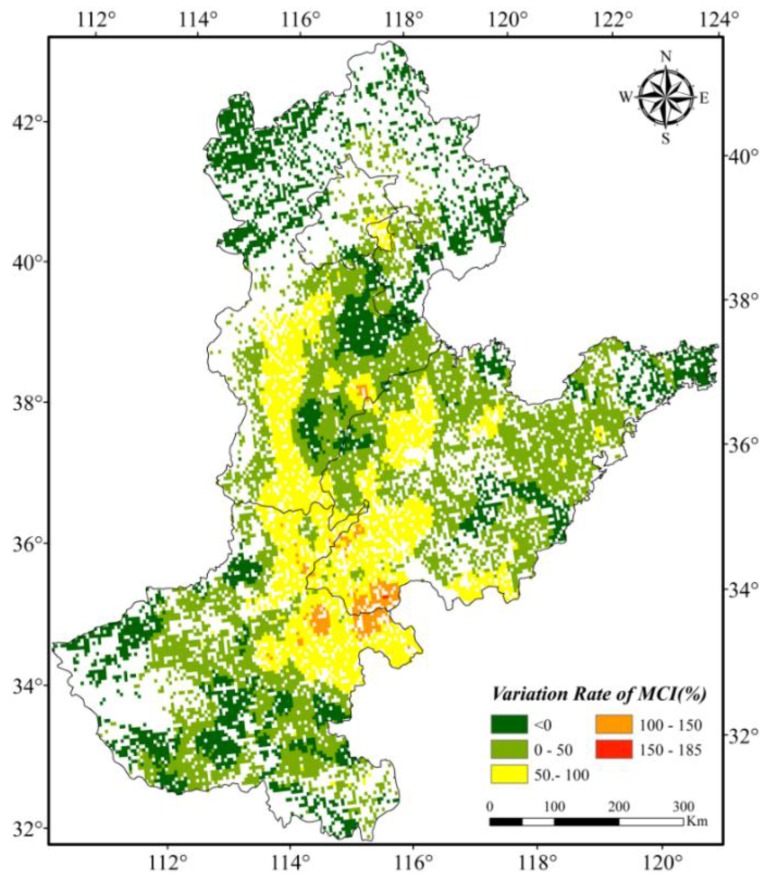
Variation rate of Multiple Cropping Indices in the North China Plain between 1982 and 2012.

**Figure 10:**
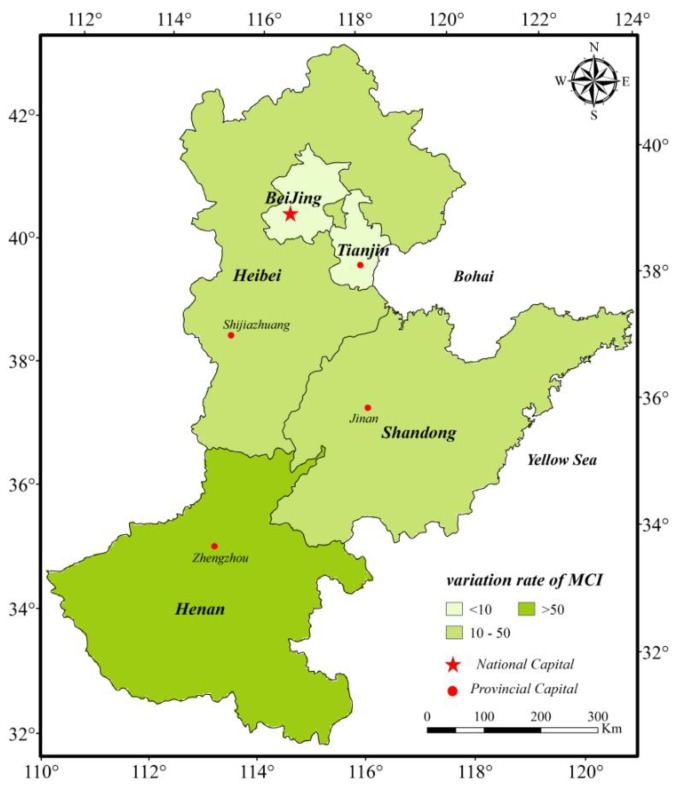
Variation rate of Multiple Cropping Indices of every city/province in the North China Plain between 1982 and 2012.

Figure 9 and Figure 10 were created based on MCI change rates to reflect the spatial distribution of MCI ranges in the subareas of the NCP. The MCI change rates of the central plain areas are greater than 50%, and they are even more than 150% in some subareas that are mostly located in the south of Hebei, west of Shandong and north of Henan (Figure 9). However, the MCI variation rates in mountainous areas, coastal regions, and parts of traditionally agricultural regions are very low, and they are less than 10% in some areas. In terms of district level, we can conclude that the MCI change rates from 1982 to 2012 in Beijing and Tianjin which are economically well developed were the least (*i.e*., less than 10%); meanwhile those of Hebei and Shandong are second, and the MCI variation rate in Henan was the highest (*i.e*., greater than 50%; Figure 10).

**Figure 11:**
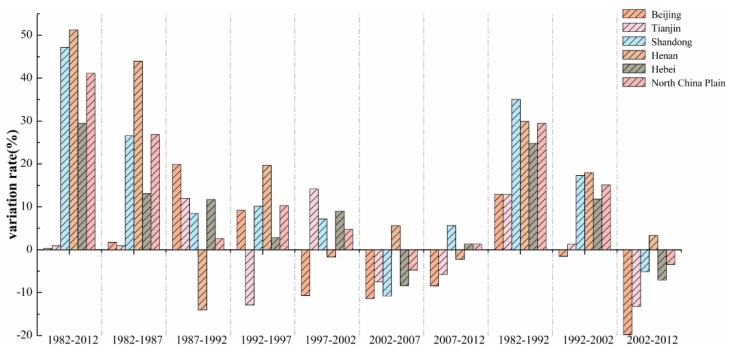
A comparison of the rate of interannual variability in the Multiple Cropping Indices of dry land in the North China Plain between the different cities/provinces for nearly 30 years.

In addition, the annual variation tendency of dry land MCI in the NCP, which was represented by the average change rate of annual MCI in this region for nearly 30 years, was not consistent. It presents a trend that the MCIs have an overall positive growth but those show a negative growth in some provinces (Figure 11). Furthermore, the partial spatial changes of MCIs of the dry land in this region had obvious geographical characteristics; for example, the change of MCIs largest, second largest, and smallest in Henan Province (whose change rate increased by 51.52%), Shandong, and in Beijing (0.32%), respectively.

### 3.2. Comparison Analysis

#### 3.2.1. Statistical Comparison

No relevant records were available from 1982, resulting in missing statistical data for Tianjin and Henan in 1982 (Table 2). Meanwhile, the accuracy of MCIs extracted by remote sensing in 1982 was abnormally low, based on the benchmark of MCI statistics in Beijing, Hebei, and Shandong, which were only about 70%; this was mainly influenced by the uncertainty of statistics and remote sensing. After 1982, the accuracy of data for each administrative region (excluding Beijing and Tianjin) was greater than 90%, meaning their accuracies were relatively high overall. As is well known, the rapid urbanization in Beijing and Tianjin has led to heavy fragmentation in farmland patches, and hence, the remote sensing images in those regions had a larger proportion of mixed pixels which would cause the lower accuracy of inverted MCIs. However, an opposite phenomenon in that the accuracy of MCIs retrieved from remote sensing data was clearly high in Hebei, Shandong, and Henan provinces was observed. This is where there was a large area of traditional agriculture with centralized farmland patches that were related to a smaller proportion of mixed pixels; therefore, the accuracy in some provinces was more than 97%.

#### 3.2.2. Comparison with the Other Remote Sensing Monitoring Results

Comparisons can be made to the results in this study with other MCIs (in 2002, 2007, and 2012) extracted from the corresponding regions based on remote sensing data with a higher spatial resolution (Table 3) [10,12,47]. This comparison shows that the various studies had generally good consistency, and the MCIs in Henan Province were the largest, but were the least in Beijing and Tianjin. In addition, our results were relatively lower in contrast with the monitoring results using finer resolution remote sensing data, and there was the largest overall difference in Hebei Province. However, the least difference was generally observed in Beijing and Henan (except the former in 2012, and the latter had the least difference of only 0.55 in 2007).

## 4. Discussion

This study analyzed the extracted dry land MCIs in the NCP over nearly 30 years based on GLASS LAI remote sensing data, as well as their dynamic spatial and temporal distribution variation. According to the MCIs retrieved from remote sensing data, we can conclude that the MCI dynamic change in this area was generated together by both natural conditions and human activities. Regarding the aspect of the natural environment, the dry land MCIs in the NCP were closely related to solar-thermal-precipitation conditions, geographical conditions, and variation of latitude in this region. Therefore, the MCIs in low latitudes with enough solar-thermal-precipitation resources and flat terrain are significantly higher than that of the high latitudes (*i.e.*, lacking in those conditions). Meanwhile, human activities also had a significant impact to the regional dry land MCIs through changing the natural conditions; specifically, the districts with more frequent human activities and higher urbanization had lower MCIs.

**Figure 12:**
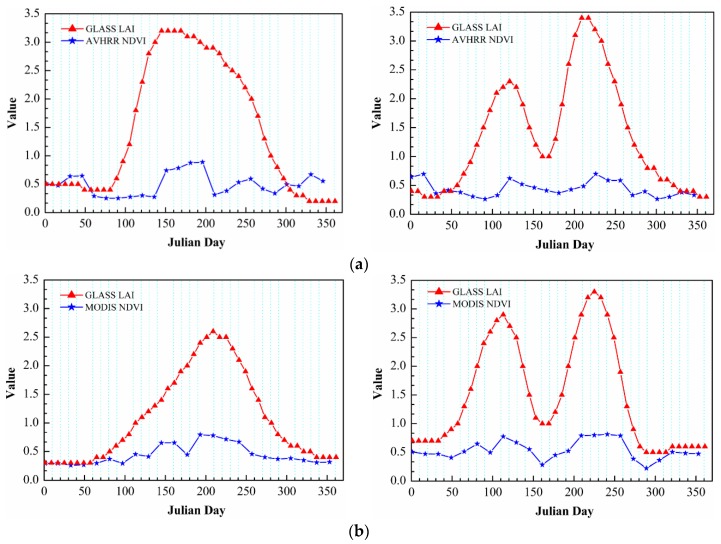
Comparing of Global LAnd Surface Satellite Leaf Area Index (GLASS LAI) with Advanced Very High Resolution Radiometer (AVHRR) and Moderate-resolution Imaging Spectroradiometer (MODIS) Normalized Difference Vegetation Index (NDVI) time series curves in the North China Plain. (**a**) The comparison between GLASS LAI and AVHRR NDVI time series unimodal (**left**) and bimodal (**right**) curves in the NCP; (**b**) the comparison between GLASS LAI and MODIS NDVI time series unimodal (**left**) and bimodal (**right**) curves in the NCP.

**Figure 13:**
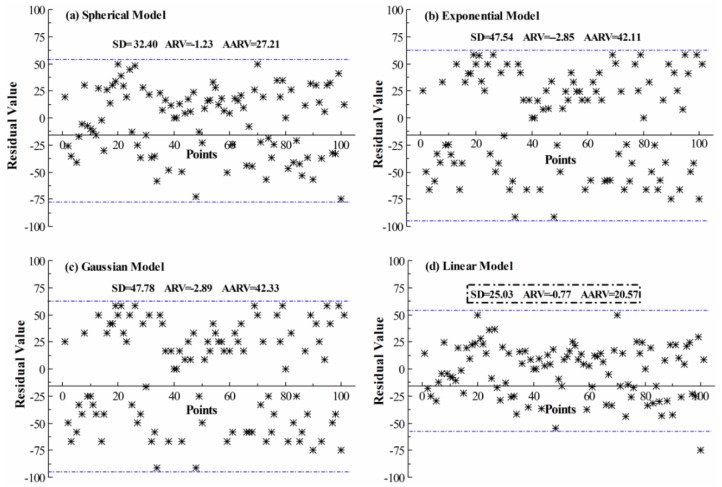
Residual value of different Kriging interpolation modes: standard deviation (SD), average residual value (ARV), and average of absolute residual value (AARV). (**a**) Spherical Model; (**b**) Exponential Model; (**c**) Gaussian Model; (**d**) Linear Model.

In this study, a few technical issues remain which need to be further explored to better understand and reduce/remove uncertainties about the MCIs being extracted by remote sensing, as follows:

(1) Compared with other vegetation index products (such as NDVI and EVI), the GLASS LAI product has a great advantage in this study. This LAI product has remarkable advantages related to the integrity of the spatial scope and continuity of time series. For example, cloud and snow spots on the images were removed, missing values were extrapolated, and the images were processed by optimized filters (see Section 2.2). Although EVI is currently a commonly used fine vegetation index, the high quality EVI product could not be obtained until 2000; therefore, it was not suitable for our research [56,57]. However, long time series NDVI products were easily obtained, e.g., AVHRR NDVI (1981–2006) and the MODIS NDVI (since 2000) dataset; nevertheless, these require elaborate consistency analysis, fusion processing, and large cross validation, *etc.* when they are synthetically used [58,59]. This shows that the GLASS LAI product is very significantly different from other aspects of valid applications. The GLASS LAI product generally has a good data quality. More pseudo peaks exist in AVHRR & MODIS NDVI time series curves than that of the GLASS LAI curves because the AVHRR & MODIS NDVI data contains a greater number of outliers (Figure 12). A considerable amount of preprocessing of the latter is thus needed to successfully extract the MCIs, which may increase the corresponding errors of extraction of MCIs and lower the availability of research results [60]. In addition, GLASS LAI data has its special merit; therefore, in this case we used the long time series LAI product to extract the MCIs of dry land over the large-scale NCP and study its cropping system.
(2) Given the fact that widely mixed pixels exist in satellite remote sensing data, the accuracy of dry land MCI extraction would be affected by the spatial resolution of remote sensing data [10,61]. In contrast, in this study, the GLASS LAI product (with a coarse spatial resolution of 0.05 × 0.05°) was used and a gridded spatial sampling strategy of selecting typical sample areas and Kriging sampling interpolation were used to obtain the MCIs of dry land in the NCP. This means that we could reduce the errors of MCI extraction to a certain extent (with the benchmark of MCI statistics in Table 2) that are derived from the spatial fragmentation of dry land in the NCP and were caused by the complicated cropping such as inter-planting and co-cropping in this region. Therefore, the correlation between the land fragmentation and accuracy of the MCIs extracted from the GLASS LAI data was not significant (Table 4). The correlation coefficients over the administrative regions are all less than 0.3. Nevertheless, the correlation coefficients in Henan and Hebei provinces were obviously only 0.066 and 0.042, respectively, but they were relatively much higher in Beijing, Tianjin, and Shandong (0.294, 0.268, and 0.263, respectively). The documented land fragmentation shows Beijing and Tianjin have higher levels of urbanization. The more complex landforms in Shandong were created by a special case with the high fragmentation of this entire region but overall with low fragmentation in local subregions. Henan and Hebei are two typical traditional agricultural regions with lower land fragmentation (Section 3.1 in detail). In particular, land fragmentation was one of the crucial factors influencing accuracies of the MCI extraction of dry land in some research, based on the higher spatial-resolution remote sensing data, where the continuously spatial research areas presented discontinuous dry land space, some patterns of nesting multi-cropping (*i.e.*, inter-planting and co-cropping) and complex topographical situations [47,55].
(3) In addition, the fact that crop LAI growth curves are reconstructed to extract the MCIs using different de-noising methods would lead to uncertainty. Currently, S-G and HANTS filters have been widely applied to smooth the crop growth curves. However, the HANTS method is often greatly influenced by setting many parameters such as frequencies, error thresholds, and number of maximum delete points. As a result, the different parameters based on different types of multiple cropping must be properly set and adjusted, especially in some areas with complicated multi-cropping structures; otherwise, the results of the curve smoothing would be affected. If the related parameters were invariably set, it could not effectively reflect the spatial-temporal real dynamics of the MCIs on a large regional scale [50,62]. In addition, the S-G filtering approach using the least square fitting method can settle the issues quite well. Consequently, in this study the more suitable S-G filtering method was employed to derive the MCIs of dry land in the NCP.
(4) The geo-statistical Kriging method was employed to map the spatial distribution and obtain the time-series dataset of the MCIs of dry land over the NCP by using spatial sampling interpolation. Owing to the different semivariogram models, they would, however, affect the results of spatial sampling interpolation and result in uncertainties of the spatial expansion of the MCIs. Thus, we quantitatively calculated the residual values between the original and interpolated data of the MCIs in the check points, based on the four types of semivariogram models (*i.e.*, spherical, exponential, Gaussian and linear models), to analyze the consistency between the source and interpolated data so as to choose the optimal Kriging interpolation model [63,64]. The residual values of the linear model are significantly lower than that of the spherical, exponential, and Gaussian models (Figure 13). The absolute values of which are almost entirely less than 50, and its data points are gathered more together with a standard deviation (SD) of only 25.03 (being compared with spherical, exponential and Gaussian models with the SDs of 32.4, 47.54, and 47.79, respectively). Additionally, its average residual value and average of absolute residual value are also the smallest with 0.77 and 20.57, respectively. This clearly indicated that the residual values of the linear model were the most stable and the sampling-expanding results derived by using it showed a good consistency with the original data. Therefore, we selected the linear model to perform a most optimal sampling interpolation expansion of the MCIs of dry land in the NCP.
(5) The reliability of the dry land area and sown area in the statistics also existed with some uncertainties to evaluate the inversion accuracy of arable MCIs in the study area [53].
(6) The validation work of this article still needs to be improved because the accuracy analysis of the research results was only implemented comparatively with the MCI statistics and previous remote sensing monitoring results, but without any verification using in-situ measurement data.

## 5. Conclusions

Based on the time-series LAI remote sensing data derived from GLASS, this study used the S-G filter and second-order difference method to effectively extract MCIs in the NCP over nearly 30 years. In addition, we analyzed its temporal and spatial change characteristics combined with a GIS (geographic information system) approach. The conclusions follow:

(1) The MCIs in the NCP were successfully extracted over nearly 30 years because the GLASS LAI data exhibited temporal-continuity and spatial-integrity. Additionally, given its advantage of less noise and further by de-noising (through the S-G filtering and smoothing method) and the reconstruction of crop growth LAI curves, it was feasible to retain the original information and extract the number of curve peaks efficiently.
(2) The MCIs in the NCP retained a stable pattern of growth overall, ranging from 100% to 155% over nearly 30 years. The MCI of dry land in 1982, 107.57%, was minimal, but the value of 152.15% in 2002 was maximal. The MCIs of this region had obvious geographical characteristics that they were high in the southern part of the study area but low in the north. Also, the MCIs in flat terrain were higher than that of mountainous and foothill areas. In addition, the dynamic variation magnitude of the MCIs also had obvious regional characteristics in that the MCI in Henan Province had a largest variation over nearly 30 years while the MCIs of Beijing and Tianjin remained relatively stable.
(3) The results were not only very accurate compared with the statistics, but also had a good agreement with several previous studies based on point experiments [10,65]. Thus, they could supplement and detail the regional spatial-temporal characteristics modes of the multiple cropping systems of dry land in the NCP to serve scientific decision making and effective management across this region. Acknowledgments: The Natural Science Foundation of China (NSFC; Grant Nos. 41171340 and 41101390), the Major International Cooperation and Exchange Project of National NSFC (Grant No. 41120114001) and the Science and Technology Innovation Program (for the Agricultural Information Institute) of the Chinese Academy of Agricultural Sciences supported this work.

## Figures and Tables

**Table 1:** Multiple Cropping Indices of different provinces (or cities) in the North China Plain (NCP) from 1982 to 2012.

Year	Beijing City	Tianjin City	Shandong Province	Henan Province	Hebei Province	NCP
1982	100.00	99.05	101.35	126.44	96.06	107.57
1987	101.72	100.00	127.90	170.42	109.12	134.43
1992	121.63	111.99	136.38	156.36	120.84	137.03
1997	130.80	99.14	146.49	176.06	123.64	147.33
2002	120.12	113.29	153.65	174.33	132.63	152.15
2007	108.75	105.79	142.88	179.95	124.24	147.40
2012	100.32	100.00	148.53	177.69	125.57	148.72
Average	111.91	104.18	136.74	165.89	118.87	139.23

**Table 2:** Comparison between Multiple Cropping Indices extracted from remote sensing data and statistics in the North China Plain from 1982 to 2012.

Year	Term	Beijing City	Tianjin City	Hebei Province	Shandong Province	Henan Province
1982	Statistical Data	151.46	--	134.86	145.10	--
	Extracted Data	100.00	99.05	96.06	101.35	126.44
	Accuracy *	66.02%	--	71.23%	70.46%	--
1987	Statistical Data	123.07	110.82	120.14	140.24	171.43
	Extracted Data	101.72	100.00	109.12	127.90	170.42
	Accuracy	82.65%	90.24%	90.83%	91.20%	99.41%
1992	Statistical Data	143.18	133.52	130.97	159.43	173.31
	Extracted Data	121.63	111.99	120.84	136.38	156.36
	Accuracy	84.95%	83.88%	92.26%	85.54%	90.22%
1997	Statistical Data	134.12	123.63	135.90	164.03	180.41
	Extracted Data	130.80	99.14	123.64	146.49	176.06
	Accuracy	97.53%	80.19%	90.98%	89.31%	97.59%
2002	Statistical Data	99.45	107.66	129.81	143.68	164.73
	Extracted Data	120.12	113.29	132.63	153.65	174.33
	Accuracy	82.79%	95.03%	97.87%	93.51%	94.49%
2007	Statistical Data	127.05	97.81	137.02	142.86	177.74
	Extracted Data	108.75	105.79	124.24	142.88	179.95
	Accuracy	85.60%	92.46%	90.68%	99.98%	98.77%
2012	Statistical Data	122.44	108.59	139.01	144.60	179.93
	Extracted Data	100.32	100.00	125.57	148.53	177.69
	Accuracy	81.93%	92.09%	90.33%	97.35%	98.75%

* Accuracy=(Extracted Data/Statistical Data)×100%, which is the same as the following.

**Table 3:** Comparison of the differences in remote sensing monitoring results of other studies in the North China Plain.

Terms	Beijing City	Tianjin City	Shandong Province	Henan Province	Hebei Province	Derived from
MCIs (%)	120.12	113.29	153.65	174.33	132.63	This study (2002)
Other results	113.4	128.2	165.3	194	153.5	Fan *et al.*, (2002) [10]
Difference value	6.72	−14.91	−11.65	−19.67	−20.87	
MCIs (%)	108.75	105.79	142.88	179.95	124.24	this study (2007)
Other results	117.8	125	169	179.4	145.1	Tang *et al.*, (2007) [12]
Difference value	−9.05	−19.21	−26.12	0.55	−20.86	
MCIs (%)	100.32	100	148.53	177.69	125.57	This study (2012)
Other results	124.5	110.6	147.6	184.3	138.6	Xie *et al.*, (2012) [47]
Difference value	−24.18	−10.6	0.93	−6.61	−13.03	

MCIs: multiple cropping indices.

**Table 4:** The correlation between land fragmentation and accuracy of extracted Multiple Cropping Indices in each subarea from 1992 to 2007.

Areas	Terms	1992	1997	2002	2007	Correlation Coefficient (*R*)
Beijing	Land fragmentation **	24.63	24.45	22.87	25.16	0.294
City	Accuracy	84.95	97.53	82.79	85.60	
Tianjin	Land fragmentation	17.25	18.86	18.10	17.81	0.268
City	Accuracy	83.88	80.19	95.03	92.46	
Shandong	Land fragmentation	23.08	24.11	22.09	23.11	−0.263
Province	Accuracy	85.54	89.31	93.51	99.98	
Henan	Land fragmentation	15.68	15.63	15.45	15.64	−0.066
Province	Accuracy	90.22	97.59	94.49	98.77	
Hebei	Land fragmentation	18.50	19.01	18.76	18.58	0.042
Province	Accuracy	92.26	90.98	97.87	90.68	

** $Land\;fragmentation=\frac{Number\;of\;land\;patches}{Land\;area}$, *i.e.*, number of land patches per ten square kilometers.
